# Determinants of Household Energy Choice for Cooking in Northern Sudan: A Multinomial Logit Estimation

**DOI:** 10.3390/ijerph182111480

**Published:** 2021-10-31

**Authors:** Philbert Mperejekumana, Huan Li, Rucong Wu, Jiaxin Lu, Obid Tursunov, Hussien Elshareef, Mohamed S. Gaballah, Nsengiyumva Jean Nepo, Yuguang Zhou, Renjie Dong

**Affiliations:** 1 Bioenergy and Environment Science & Technology Laboratory, College of Engineering, China Agricultural University, Beijing 100083, China; philbertson2@yahoo.com (P.M.); huanli828@cau.edu.cn (H.L.); wu_rucong@163.com (R.W.); sltanmka@gmail.com (H.E.); rjdong@cau.edu.cn (R.D.); 2 Key Laboratory of Clean Production and Utilization of Renewable Energy, Ministry of Agriculture and Rural Affairs, Beijing 100083, China; 3 National Center for International Research of BioEnergy Science and Technology, Ministry of Science and Technology, Beijing 100083, China; Saadga22@gmail.com; 4 School of Ecology and Environment, Beijing Technology and Business University, Beijing 100048, China; lujiaxin@btbu.edu.cn; 5 Department of Power Supply and Renewable Energy Sources, Tashkent Institute of Irrigation and Agricultural Mechanization Engineers, Tashkent 100000, Uzbekistan; obidtursunov@gmail.com; 6 Research Institute of Forestry, Tashkent 111104, Uzbekistan; 7 Department of Agricultural Engineering, College of Agriculture, University of Khartoum, Khartoum 11115, Sudan; 8 Department of Marine Environment, National Institute of Oceanography and Fisheries, NIOF, Alexandria 21556, Egypt; 9 College of Agriculture Animal Science and Veterinary Medicine, University of Rwanda, Kigali 4285, Rwanda; jeannepo40@gmail.com

**Keywords:** cooking fuels, multinomial logit model, biomass, firewood, household’s choice, biomass, pollution

## Abstract

Traditional biomass utilization is connected with negative environmental and human health impacts. However, its transition to cleaner cooking fuels is still low where the household’s fuels preferences play an important role in the process. To examine the factors that influence the household’s cooking fuel choice in Northern Sudan, a multinomial logit model (MNL) was used to analyze data collected from Kassala state in two selected districts, New Halfa and Nahr Atabara. The findings show that the most utilized fuels are still firewood and charcoal, which are used by 63.4% of all respondents. The results also revealed that socioeconomic factors have an impact on household fuel choice, where one additional unit of credit access may boost the possibility of choosing LPG by 22.7%. Furthermore, one additional level of education would reduce 5.4% of charcoal users while simultaneously raising 10% of current liquefied petroleum gas (LPG) users. Therefore, the study suggests initiating mobilization and training programs to raise awareness and encourage the usage of cleaner fuels. This study will provide policymakers with information on household cooking energy utilization while designing and developing policies related to energy. It will also contribute to the expanding body of literature concerning the transition to clean cooking fuels from traditional biomass.

## 1. Introduction 

Cooking activities consume roughly 90% of the energy consumed by households in developing countries, making cooking fuel choices a major concern for both households and policymakers [1]. Numerous studies have shown that the cooking energy choice warrants consideration because relying heavily on non-clean energy (mostly biomass and coal) has significant health and environmental consequences [2,3,4,5,6]. In addition, there are other numerous consequences connected with the use of inappropriate cooking fuels and stoves amongst developing countries. Regarding this point, solid fuel combustion with an ineffective cookstove has been found to cause more premature deaths than HIV/AIDS, malaria, and tuberculosis combined, with many deaths occurring among females and children under the age of five [3,4,5,7,8,9]. According to several researchers, approximately 3 billion people use solid fuels such as wood, charcoal, animal dung, and crop residues to cook, heat, and/or light their homes, with nearly half of them using inefficient, poorly ventilated stoves [10,11,12,13], in Africa in particular, more than 82% proportion use solid fuels [14]. There is an expectation that due to the rapid population growth rate in rural areas, the number of biomass users will rise to 2.7 billion by 2030 [2]. Different works in the literature mentioned that the use of traditional cooking methods is extremely high especially in developing countries where, according to Uttam Paudel et al. [15], solid fuels are used by more than 2.5 billion people. In Sudan, charcoal is the primary cooking fuel for more than 89% of urban households, while firewood is the primary cooking fuel in rural areas, accounting for more than 81 percent of households [16]. According to FAO [17], Sudan has been struggling to provide people with affordable and long-lasting fuel. Among the reasons are the following: (a) a heavy burden for women and girls who must travel up to 13 kilometers three times a week to collect fuelwood; (b) unsustainable exploitation of forest resources, resulting in increased conflict over the limited forest and tree resources; (c) high cost of fuelwood and charcoal; (d) the health and safety hazards of cooking over a conventional three-stone fire. Despite these limitations, Sudan has made a firm commitment to continue research, design, and implementation of new energy technologies, with expanded access to safe and reliable energy resources stimulating new development options, as well as sustainable low-carbon energy scenarios for the twenty-first century that emphasize renewable resources’ untapped potential [18].

Therefore, according to the available literature, in Northern Sudan there is a need for widespread implementation and use of advanced, renewable, and clean energy sources [13,17,19]. This is in line with the 2030 United Nations Sustainable Development Goals (SDGs), especially Goal 7: "Ensure universal access to affordable, secure, sustainable, and modern energy for all by 2030" [14]. Many citizens, however, find it difficult to switch to alternative energy sources because the transition from traditional to clean cooking methods is still restricted in many countries. According to Uttam Paudel et al. [15], strong regulation, standards, investment, and policies all help to promote and encourage a rapid transition from conventional cooking to clean fuel technology. Therefore, numerous studies have been conducted to identify possible determinants that affect household cooking fuel choices and their results revealed that demographic and socioeconomic factors are among the determinants that influence the households cooking energy choice [1,2,4,15,19,20]. However, renewable energy usage, according to the majority of the researchers’ results, would help to minimize energy shortages and increase energy efficiency, as well as reduce energy-related negative impacts [15,21]. Therefore, many studies have looked into the factors that affect the utilization of modern and traditional fuels, and they have found that household income, education, and gender of the household head, household size, fuel prices and availability, and location all play a role [15,19,21,22,23,24]. Moreover, some studies say that due to expense, culture, and fuel supply reliability, adopting improved cookstoves and modern fuels would be nearly impossible for some socioeconomic groups in the immediate future [4]. A study conducted by Yonas et al. [24], about modeling household cooking fuel choice in Ethiopia by using the multinomial logit model (MNL), found that households’ economic status, price of alternative energy sources, and education are important determinants of fuel choice. In addition, Nnaji et al. [21] conducted a study on the determinant of household energy choices for cooking in Nigeria by using the same model and found that income is the important factor that determines the household’s cooking fuel choice. 

However, the study carried out by Ahmed [16] in Northern Sudan, revealed that the price of conventional biomass fuels versus LPG, when available, is a significant determinant factor in households’ decision to switch to new energy technology. Thus, the recent research article aimed to furthermore shed light on factors that influence household cooking energy choices in Northern Sudan and to explain how households choose cooking fuel in the face of uncertainty or when transitioning to modern fuels. To attain that goal, a household survey was conducted from two districts of Northern Sudan, namely New Halfa and Nahr Atabara, randomly selected from Kassala state. The majority of the population in both provinces’ economies is based on agriculture and a great proportion of them use charcoal and firewood to meet their cooking needs. Corrected data have been analyzed by using STATA and SPSS through a multinomial logit model (MNL) to identify and analyze key factors of household cooking energy choice and forecast the outcomes with the following objectives: (1) To identify factors that play a role in the household’s cooking energy decision-making; (2) to investigate the probability of the transition from traditional to modern cooking energy at household level; (3) to discuss the possibilities of the households’ adoption of clean cooking technology based on the findings. The empirical results can be a tool to guide policymakers while designing policies regarding household-level cooking energy. The findings could aid future researchers in this field. Moreover, data provided in this paper may help energy-related investors to recognize the direction of their future investment. 

## 2. Literature Review: Conceptual Analysis 

Several studies on cooking energy at the household level in developing countries have shed light on the determinants that influence a household’s decision to choose a particular cooking fuel. The limited but rising empirical literature on energy choice offers little insight into the factors that influence household fuel choice and switching behavior through a lens of the econometrics model [24]. Moreover, in light of energy transition among developing countries, the determinants that influence clean cooking energy (wood pellets, LPG, etc.) choice at the household level are not clearly identified and there is still a lack of knowledge. However, recent studies from various countries have found that cooking fuel use decisions are influenced by several demographic, social, and economic factors using both descriptive and quantitative approaches. Theoretically, those factors in light of energy demand were found to be in a non-linearity function relationship. In this regard, a variety of techniques have been applied such as logit/probit. A multinomial logit model is one of the foremost models for analyzing the determinants that influence a household’s decision regarding energy choice. 

Notably, some studies on the determinants of household energy choice in the literature often lack sufficient data, especially on the traditional biomass utilization to cleaner cooking fuels. Among such studies include Amin Karimu et al. [4] and Twumasi et al. [2] in Ghana; Yonas Alem [24] in Ethiopia; Gould et al. [22] in India; Nnaji et al. [21] in Nigeria; Boukary [25] in Burkina Faso. In some regions, cooking fuel options are a little more complicated because modern fuels are frequently used alongside traditional biomass fuels in some families. Evidence from Ahmed Ado [26] using a multivariate analysis reveals that low-income and often uneducated households use traditional fuels, whereas the middle and upper class of society primarily use transitional and modern fuels. Households choose multiple fuels for a variety of reasons, including the fact that entire reliance on a single fuel may be sensitive to price changes and unreliable service/supply, as well as experience with cooking using traditional methods [25]. This is consistent with Ifegbesan et al. [27], who came to the conclusion that firewood is the primary source of energy for the majority of Nigerian homes, especially in rural areas. 

Nevertheless, traditional energy sources such as fuelwood, charcoal, dung, and crop residues dominate in energy consumption of Africa and remain a key constraint to the transition of households to modern and clean fuel. Those traditional energy sources were found to be connected with negative ecological and health impacts [24]. A number of studies have found that a high reliance on traditional solid fuels has raised global concern about both the harmful health effects such as tuberculosis, lung cancer, and respiratory infections; and the environmental repercussions of indoor air pollution such as forest degradation and soil erosion [8,28,29,30,31]. Therefore, the take-up of modern fuels and improved stoves are solutions to these consequences which are still at a low stage especially among African countries [32,33,34]. To do so, a better understanding of the factors that influence household cooking energy choice is required. 

In terms of the methodological approach, the available literature are varied based on parametric aspects including (logit and probit), multinomial probit and logit, full information maximum likelihood (FIML) techniques, and the ordinary least square approach. For example, Paudel et al. [15] employed a multinomial logit model (MNL) to investigate the pattern of household cooking fuel usage and the association of household cooking fuel choices. However, the use of an MNL for estimating those factors influencing a household’s cooking energy choice is very scanty in the extant literature and completely non-existent in the North Sudan context. Therefore, this paper makes a contribution to this by understanding the factors influencing a household’s cooking energy choice and it is expected to have significant input in addition to past research’s conclusions, which will aid in encouraging the switch to clean cooking fuels as well. 

## 3. Materials and Methods 

### 3.1. Data Collection and Sampling

Data collection was carried out by using a designed questionnaire from December 2020 to February 2021 which was followed by an interview in order to enhance the significance of the study. The scholars, local farmers, and traders were interviewed to verify whether the collected data was logical and goal oriented. During the questionnaire design, measurement tools have developed where we first created a list of clean cooking energy adoption and utilization indicators based on the literature review. During the first stage, the list contained 50 indicators but after being reviewed by a committee of experts in the field of cooking energy, including professors and scholars, it was reduced to only 25 indicators, which were used for the examination. The questionnaire was designed with three main sections. In the first section, the respondents had the option to choose corresponding basic information about their location, gender, age, and education level. The second section was designed with multiple-choice questions indicating monthly income and cooking expenditures of the household, type of fuels and stove, people in their family, and occupation. Section 3 investigates the factors affecting respondents’ readiness to choose their primary cooking fuels and how they contribute to policies of national new energy and environmental protection. A simple random sample method was used, in which all participants were given an equal chance to respond to the questionnaire that was distributed in the study area, where two districts, New Halfa and Nahr Atabara, were chosen from one province, Kassala in Northern Sudan. Agriculture is the main financial source in both districts and the majority of the population uses traditional fuels for cooking. Four sectors were chosen at random from each district, to make a total of 8 sectors. Then, 35 households from each sector were chosen at random to participate in the study, with a total of 280 participants. Only one questionnaire was deemed irrelevant and the remaining 279 were found worthy of analysis and hence used in this study. 

### 3.2. Methodology 

The collected data was analyzed using SPSS (SPSS Inc., Chicago, IL, USA) and STATA (Stata Corp LLC, TX, USA) by employing a multinomial logit model to identify the factors that influence households’ cooking fuel choices. During the model running, multinomial logistic regression was used to examine the determinant driving the cooking energy choice among households in Northern Sudan. The multinomial logit model, on the other hand, is appropriate when the household has a large range of highly desirable options. According to our findings, the majority of households utilize charcoal, wood fuel, and LPG as their primary sources of cooking fuel, with wood, straw/grass, animal dung, and other materials coming in second. Therefore, the multinomial logit model was found to be suitable for addressing the objectives of this study. The focus of this study was entirely on the analysis of household energy probability options for the first three most widely utilized primary fuels, namely LPG, charcoal, and firewood. It is expected that the likelihood of one alternative choice being picked over a second is unchanged or unaffected by the absence of a third option [35]. In addition, the quantity desired of an item is negatively influenced by its price and accessible replacements and positively influenced by population, according to the demand theory established by Uttam Paudel et al. [15]. Therefore, LPG, electricity, kerosene, and coal can be used as substitutes for wood and animal dung fuel. The definition of explanatory variables employed in this study’s analysis are presented in Table 1. 

### 3.3. Multinomial Logit Model

The multinomial logit (MNL) model was used to determine the factors that influence cooking fuel choice. Tsourgiannis et al. [35] used the same model to determine the empirical relationship between cooking fuel and factors hypothesized to impact the decision. The goal of the model is to show how changes in the predictors translate into the likelihood of seeing a specific category outcome. The multinomial logit model is appropriate because it identifies statistically significant correlations between explanatory variables, such as socioeconomic, institutional, and physical factors, and a dependent variable (fuel cooking choice). MNL, unlike other models such as log-linear regression and discriminant analysis, does not rise by a constant amount but approaches zero at a slower rate when the value of an explanatory variable decreases. It can also be employed when a mixture of numerical and categorical variables are present. The MNL model is defined using the aforementioned information, with market choice as follows:

(1) $$CKINGFCH_{ij}=\beta_{j}+X_{ij}+\in_{ij}$$

where *CKINGFCH_ij_* is a vector of the 3 cooking fuel choices of *i*th household, *β_j_* is a vector of fuel choice-specific parameters, *Є_ij_* is the error term assumed to have a distribution with mean 0 and variance 2, *X_ij_* is a vector of the household’s characteristics that together reflect the incentive, risks, and capacity variables and other shifters influencing the household’s indirect utility. If the household chooses cooking fuel *j*, then *U_ij_* is the maximum among the *j* = 1, 2, 3 utilities. It follows that if cooking *j* will be chosen by a household, then

(2) $$PROB\left(U_{ij}>U_{ik}\right)\;\;\;\;\;\;\;\;\;\;\;\;\;\;\;\;\;\;\;\;\;\;\;\;\;\;\;\;\;\;\;\;\;\;for\;k\neq j$$

Following Greene [36], the probability for the choice of cooking energy type *j* given *x_i_* covariates is given as:

(3) $$PROB\;\left(Y_{i}=j\right)=\frac{e^{\beta_{j}x_{i}}}{1+\sum_{i=1}^{i=n}e^{\beta_{j}x_{i}}}$$

where *Y_i_* is the market choice *j* made among a total of different sources of cooking energy by households *i*, *x_i_* is the household level and area-specific factors of choice of the household *i*, and *β_i_* is the parameters to be estimated. Specifically,

(4) $$PROB\;\left(Y_{i}=j\right)=\frac{1}{1+\sum_{i=1}^{i=n}e^{\beta_{j}x_{i}}}$$

The parameters can be estimated by the maximum likelihood procedure as:

(5) $$Ln=\left[\frac{P_{ij}}{P_{i1}}\right]=\beta \prime_{j}x_{i}$$

where the dependent variable is the log odds that the household will choose cooking fuel *j* relative to the base category. The marginal effects are then estimated to show the probability for the ranking between 1 and 3 for a given cooking fuel by:

(6) $$\frac{\partial p}{\partial x_{i}}=\frac{\partial }{\partial x_{i}}\left[\exp\left(x^{\prime }\beta \right)/(1+exp\left(x^{\prime }\beta \right)\right)]=p\left(1-p\right)\frac{\partial x^{\prime }\beta }{\partial x_{i}}$$

It should be noted that the base outcome determines simply the model’s parameterization, not the chance of household *i* choosing cooking energy type *j*. The empirical model for estimating the relationship between cooking fuel and influencing factors was defined as follows:

(7) $$CKINGFCH=\beta_{0}+\beta_{n\;}(socioeconomic;\;institutional,\;cost\;factor)+Uij$$

where the variables are defined in Table 1 and *U_ij_* is the error term. Consequently, during the research, families were classified into three mutually exclusive groups depending on the types of cooking fuel used, such as (i) charcoal, (ii) firewood, and (iii) liquefied petroleum gas (LPG). A multinomial logit model was used to investigate the factors that influence households’ choice of cooking fuel in Kassala Province of Northern Sudan. This model was chosen because of its superior performance in discrete choice studies [18]. Each household’s socioeconomic and demographic variables (household size, education level, household size, gender, income, location, cooking expenses, and so on) will impact the choice of a cooking fuel source, and these characteristics will differ from one household to the other [25]. Therefore, the possibility of a household choosing one type of cooking fuel ranges from zero to one. The alternative set contains no reallocation, and the model assumes no changes in fuel costs or fuel qualities. Furthermore, the model implies that households select fuels that maximize utility [21]. 

## 4. Results 

### 4.1. Descriptive Results 

Table 1 represents the definition and descriptive statistics of all variables used in the study analysis. The results revealed that 58.4% of the total respondents are households that have between 1 and 6 members. In addition, among households that participated in this study, 49% were male while 45.3% were female. However, the results also show that 63.9% confirmed that females are the ones who cook in the family. Furthermore, regarding the distance from the household to the source of cooking fuels, only 13.9% get cooking fuel less than 2 km from their homes, while 80.4% get the fuel from 2 km or more. However, their preference fuel decision on cooking fuel if the choice is given had been made clear, where around 50% of traditional biomass users confirmed that if they are given a choice, they prefer to choose LPG as their primary cooking fuel. Regarding criteria followed by households while choosing fuel, 30.7% of the respondents confirmed that they base their decision on the fuel cost, 22.6% on fuel availability while 15.4%, 13.06%, and 14.3% base their decision on the fuel that emits less smoke, the quantity of fuel per meal, and time saving respectively. Furthermore, the results presented in Table 2 show that 52.3% of the respondents that participated in this study are male, while 47.7% were found to be female. 

### 4.2. Cooking Fuel and Occupation 

**Figure 1:**
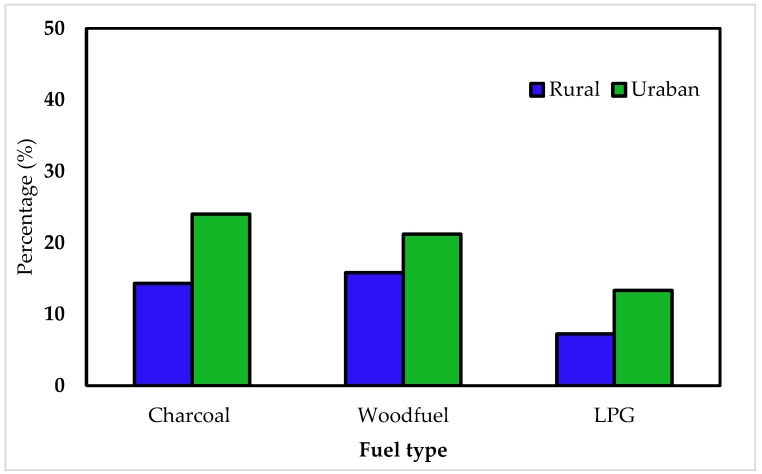
Primary cooking fuel by location.

The participants cooking fuels patterns and occupation are presented in Table 1, where according to the results about 24.4% of the total sampled households mainly cook with charcoal, while 14.3% mainly cook with firewood especially in rural areas (Figure 1), and 24.7% with charcoal and firewood sometimes. This implies that 63.4% of the total respondents that participated in this study use charcoal and firewood for cooking. It is worth noting that charcoal and firewood would be treated differently as energy sources because households who prefer charcoal have different characteristics (apartment size, monthly income, family size, and education level) than those who prefer firewood. Moreover, 4.3% mainly use coal while the remaining 32.3% use LPG. In addition, the occupation distribution among households that contributed to this study shows that 21.5% are civil servants, 32.6% are farmers, 33.7% are traders, and only 11.8% are self-employed (Table 2). Furthermore, Figure 1 shows that, despite the fact that rural households use less charcoal than urban households, they also use more firewood with 14.34% of the overall respondents using charcoal, 18.64% using wood fuel, and 7.89% using LPG. Charcoal and LPG appeared to be used more frequently in urban areas than in rural regions, with 25.1% and 13.6% of urban residents using charcoal and LPG, respectively, and 20.4% utilizing firewood for cooking.

### 4.3. Size of Apartment and Education Background 

**Figure 2:**
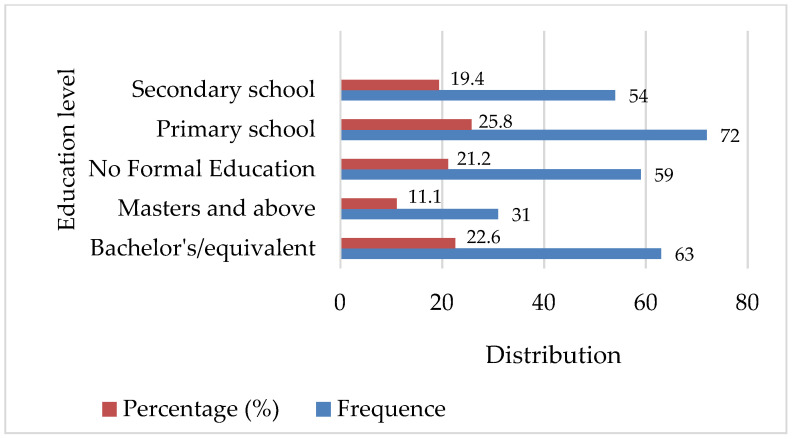
Household’s educational background.

Figure 2 shows that 22.6% of the total respondents that participated in this study have a bachelor’s/equivalent, while 11.1% have a masters and above which implies that 33.7% of the total respondents have attended university. In addition, 25.8% of the total respondents also completed primary school and 19.4% completed secondary school. This means that up to 78.9% attended the school from primary and above while the remaining 21.2% have no formal education. Moreover, respondents have been asked which kind of fuel type they would prefer if they are given a choice and their responses are presented in Table 2 where the majority of them chose LPG as the preferred fuel. Approximately 49.5% of respondents who participated in this study said they would prefer to use LPG if the choice is given. If given an option, 14.3%, 20.1%, 6.5%, and 6.7% of the respondents stated that they would choose charcoal, firewood, wood pellets, and coal respectively. In addition, the results show that 66.3% of the total households that participated in this study live in apartments with less than 4 rooms, while 33.7% live in those with 4 rooms and above (Table 2). 

### 4.4. Clean Cooking Fuels Awareness 

The findings in Table 2 and Figure 1, which show that a substantial number of households utilize traditional fuels instead of clean cooking fuel, are compatible with the results presented in Table 3, which show that 26.5% of households are unaware of clean cooking techniques. Furthermore, 3.9%, 28.3%, and 23.7% received information through the internet, radio, and television, respectively, but only 17.6% met with a mobilizer who explained the clean cooking technique. Moreover, 72.6% of the total respondents who participated in this study have no training about clean cooking equipment usage, where only 22.9% of them are trained. 

### 4.5. Econometric Findings

The factors that affect the household’s cooking fuel option in Northern Sudan were identified using a multinomial logit model (MNL) with firewood as the base outcome. Following that, the multinomial logit model reported that eight (8) variables out of twenty-nine (29) explanatory variables determined the probability of households choosing charcoal as a fuel source, thus fifteen (15) variables out of twenty-nine (29) explanatory variables were discovered to assess the probability of households’ decision to use liquefied petroleum gas (Table 4). The impact of minor changes in the important explanatory variables on each cooking fuel is addressed in greater detail below.

The McFadden pseudo-*R*^2^ was chosen for the simplicity of its calculation. Based on the likelihood ratio, the *R*^2^ by McFadden is as follows:

(8) $$R^{2}=1-\frac{Log\left(L_{UR}\right)}{L_{R}}$$

where $L_{UR}$ is the maximum of the likelihood function of the model without constraints, and $L_{R}$ is the maximum of this same function by forcing the coefficients of all exogenous variables to be zero. Mainly, *R*^2^ commonly called the coefficient of determination, is a measure of how well a linear regression model fits a dataset. Therefore, researchers evaluate their models based on it, and always the higher value is better to explain changes in the outcome variable. According to Cohen [37], an *R*^2^ value of 0.12 or below indicates low, between 0.13 to 0.25 values indicate medium, 0.26 and above and above values indicate high effect size. In our case, McFadden’s pseudo-*R*^2^ gives *R*^2^ a value of 0.26, suggesting that exogenous variables selected to be important account for 26% of the energy choices of the study area’s households. This pseudo-*R*^2^ value indicates that the model is reasonably efficient. 

## 5. Discussion 

### 5.1. Factors Influencing Cooking Fuel Choice 

#### 5.1.1. Location and Education Level 

The findings show that urban households have a positive and significant impact on charcoal fuel choice, while the impact on LPG fuel choice is positive but not significant. This indicates that in the study area, people in urban areas prefer charcoal to LPG (Figure 1), which is consistent with a report by the United Nations Environment Programme (UNEP) [16] that mentioned that Khartoum has the highest urban charcoal consumption per capita. Education level (Figure 2) was also found to be statistically significant at a 10% level of significance and to have a negative effect on the choice of charcoal while having a positive impact on the choice of LPG and being statistically significant at a 5% confidence level (Table 3). This implies that a one-unit increase in education will reduce 5.4% of charcoal users while simultaneously causing a 10% increase in current LPG users. Different studies have reported similar results, confirming that as the head of the household’s education level rises, so does the household’s perception of fuel market trends, immediately rising information and awareness about new cooking technologies [24,25]. This is consistent with the research conducted by Dagnachew et al. [9] who found that people with higher levels of education are more likely to embrace new cleaner fuels. Furthermore, according to the research conducted by Uttam Paudel et al. [15] in Afghanistan, the education level of the household head is more likely to be correlated with the probability of choosing LPG and wood over biomass fuel.

#### 5.1.2. Who Cooks in the Family 

As expected, our findings revealed that women’s share has a positive but non-significant effect on charcoal fuel choice, while it has a positive effect on LPG fuel choice and is statistically significant at the 5% level of confidence. This means that increasing the female share of cooking activity in Northern Sudan will result in a 9.2% increase in the population using LPG. Females are more likely to adopt and use LPG than charcoal, according to a recent survey. There are a number of explanations for this, including the fact that they are in charge of gathering fuel and cooking [17]. Therefore, they choose LPG because it is safer and more convenient for them so that they do not have to deal with the drudgery of fuel collection. This is consistent with the findings of Yonas Alem et al. [24] who found that females in the household tend not to use biomass fuel because it takes up a considerable amount of time and could have a greater negative effect on their health because combustion of such conventional fuels can cause indoor pollution.

At a 5% level of confidence, our estimation findings also indicate that any member of the family who participates in cooking is positively and statistically significant. This may be due to the fact that if one family member does not know how to use LPG equipment, any family member who does would intervene, increasing the likelihood of the household choosing LPG by 13.1% compared to existing users if increased by one unit. 

#### 5.1.3. Acquaintance with Clean Cooking Fuels and Equipment 

Furthermore, training on how to use LPG equipment was found to have a positive impact on the household’s LPG option with a 5% degree of significance, according to the findings. As a result, if an additional unit of any family member is trained with the use of LPG equipment, the probability of LPG adoption and use will increase by 9.3% for current users. This increase in probability is due to the qualified people gaining enough information about how to use the burner, along with visual aids (not turning the heat regulator knob all the way up, as this may burn the food), lighting, and temperature control to cook a variety of food types, which could encourage them to switch to LPG. This is in line with the findings from the research carried out by Jagger and Das [33], who revealed that providing customers with training on how to use modern cooking technology equipment is essential for safe and successful use.

As expected, cooking time per day was found to have a negative impact on both charcoal and LPG household preference, with a 10% and 5% level of significance, respectively. This means that adding one unit to cooking time per day will reduce household preference for charcoal and LPG by 5.8% and 12.4%, respectively. This is due to the fact that most households prefer fuel that does not require them to spend too much time cooking in order to free up time for other essential activities. That is similar to the results of Lim et al. [38] and Laufer et al. [39] who discovered that men and women in China, Indonesia, and Sri Lanka save time by using modern energy for different purposes. Men, for example, use it for relaxation and entertainment, while women use it for a variety of purposes, including earning money, doing housework, spending time with their children, and relaxing. Choosing cooking fuels based on those that emit less smoke had a 5% level of significant positive effect on the household’s choice of charcoal but had a 10% level of significant negative effect on the household’s choice of LPG. This indicates that adding one unit to households that choose fuel based on which emits the least amount of smoke will increase the likelihood of choosing charcoal by 11.5% while decreasing the likelihood of choosing LPG by 13%. This is because low-income households are more likely to choose those that emit less smoke. As a result, they cannot use charcoal or LPG, but they may use firewood, agricultural residues, or animal dung. These findings are consistent with the study conducted by André Paul Neto-Bradley et al. [40] on low-income households in Bangalore, who discovered that all households surveyed use firewood and that they all use multiple fuels, with just over 10% of them using LPG for cooking. 

#### 5.1.4. Financial Status and Household’s Size 

Furthermore, our findings revealed that monthly household income has a positive impact on both charcoal and LPG household preference, with a 5% level of significance on both sides. This means that for current consumers, an increase of one unit in monthly income would increase the likelihood of choosing charcoal by 6% while raising the likelihood of choosing LPG by 7.6%. The explanation for this is that LPG is prohibitively costly for low-income households, as a result of their financial constraints, they tend to use locally available fuels like firewood, charcoal, and agricultural residues (Table 2). However, when a household’s monthly income rises from low to high, they tend to use clean cooking technologies, according to the findings. These findings are in line with those from the study of Baland et al. [41] who also came to the conclusion that household income affects household willingness to adopt new cooking devices. Moreover, Amin Karimu et al. [4], who conducted a study on LPG adoption in Ghana, also found that income is one of the most significant factors influencing the decision to use LPG for cooking. However, the multinomial logit model revealed that credit usage has a positive influence on both charcoal and LPG household decisions, with a 5% and 1% degree of significance, respectively (Table 3). This means that increasing the number of households with access to credit by one unit increases the likelihood of choosing charcoal by 7.2% while increasing the likelihood of choosing LPG by 22.7%. This shows that if low-income families have access to credit, they would be able to use healthier cooking fuel. The results of the current work are consistent with the research carried out by Twumasi et al. [2] who portrayed credit as a critical tool for rural households to choose and spend on clean cooking energy. Van Rooyen et al. [42] and Atieno et al. [43] found similar results suggesting that credit has a positive impact on clean cooking adoption. Furthermore, a number of previous studies have shown that credit increases household patterns and per capita income, which has a direct effect on conventional fuel consumption by enabling households to buy clean cooking technologies [44,45]. The size of the apartment was found to have a positive impact on the household’s choice of charcoal but was not statistically significant, while the size of the apartment had a positive impact on the household’s choice of LPG and was statistically significant with a 5% level of confidence. This means that increasing the size of a household’s apartment by one unit raises the likelihood of using LPG by 10.1%. In Northern Sudan, this means that as the size of the apartment expands, households are more likely to follow clean cooking technologies.

## 6. Conclusions 

The negative effects on ecology and health connected with the use of traditional cooking fuels push countries in working hard to transition to sustainable cooking technologies through a variety of strategies, such as promoting improved cookstoves and integrating clean cooking fuels. However, selecting the best cooking method when encouraging energy change necessitates a deeper understanding of the factors that affect household energy choices. In this regard, information about the factors that affect household decisions in Northern Sudan is still limited based on available studies, which is why more empirical evidence would aid in the development of effective energy and environmental strategies. Therefore, the main purpose of this study has been to investigate the determinants that influence cooking energy choice and see how well they explained the observed behavior of household energy choices in Northern Sudan. The multinomial logit model has been used to identify the determinants of energy for cooking as well as sociological and economic variables influencing major energy sources in the study area. The results depicted that variables such as location, household size, size of the apartment, education level, and criteria followed by the household for choosing cooking fuels are the crucial determinants for fuel choice. Furthermore, the findings displayed the importance of a household’s monthly income, credit use, and training about modern cooking approaches and equipment in switching from traditional fuels to clean cooking. This study suggests that all these variables should be taken into consideration by policymakers while addressing issues regarding household energy. Furthermore, one solution to the environmental implications of unsustainable wood exploitation is to make modern cooking fuels more available and affordable, as well as to make the use of firewood and charcoal more sustainable. In addition, proper policies should be designed to educate and train a large number of people in order to enhance the likelihood of households expanding their usage of alternative fuels. Finally, since the majority of people prefer firewood and charcoal as fuels, and because the transition to new and renewable energy could be sluggish and indirect, the reforestation strategy should be improved to resolve energy demand issues as well as other relevant problems such as soil erosion and desertification.

## Figures and Tables

**Table 1:** Definition and descriptive statistic summary of variables used in multinomial logit regression.

Variable	Description	Mean	Std. Dev.
Location	Where do you live? (0 = rural, 1 = Urban)	0.591	0.492
HH Size	Household has 1–6 members? (1 = yes, 0 = no)	0.620	0.486
Education Level	The level of education of the head of the household (high school and above) (1 = yes, 0 = no)	0.484	0.501
Migration	Migrated here? (1 = yes, 0 = no)	0.710	0.455
Fuel Type	Which fuel do you use? (0 = charcoal, 1 = wood fuel, 2 = LPG)	0.821	0.761
Distance Market	Is the distance from your household to near the fuel market 2 km and above? (1 = yes, 0 = no)	0.853	0.355
Gender	What is your gender? (0 = male, 1 = female)	0.480	0.501
Who Cooks	Who cooks in your family? (0 = house maid, 1 = any family member, 2 = female, 3 = male)	1.581	0.662
Cooking Time	Do you spend 4 hours and above on cooking per day? (1 = yes, 0 = no)	0.197	0.399
Kitchen	Do you have a kitchen outside the main house? (1 = yes, 0 = no)	0.563	0.497
Criteria	What are your criteria to choose fuel? (0 = cost, 1 = availability, 2 = smoke, 3 = quantity 4 = time).	1.531	1.429
CC Info	Where did you hear about clean cooking? (0 = radio, 1 = television, 2 = mobilizer, 3 = friend, 4 = no info)	2.376	1.540
Credit Use	Do you have access to credit? (1 = yes, 0 = no)	0.373	0.484
Monthly Income	What is your family’s average monthly income? 19844 and above? (1 = yes, 0 = no)	0.455	0.499
Cooking Expenc	What are your monthly expenses on cooking fuel? 1984 and above (1 = yes, 0 = no)	0.387	0.488
Size of Apart	What is the size of your apartment? (number of rooms)? 4 and above (1 = yes, 0 = no)	0.348	0.477
Training	Did you get any kind of training about how to use clean cooking equipment? (1 = yes, 0 = no)	0.229	0.421
Prospected Choice	If you were given fuel choice, what would it be? (0 = LPG, 1 = charcoal, 2 = firewood, 3 = pellet, 4 = coal)	1.125	1.347

Note: In the table, HH size—household size, CC info—cean cooking information source, Cooking expenc—cooking fuel monthly expenses, Size of apart—size of apartment, and Std. Dev.—Standard deviation.3.3. Multinomial Logit Model.

**Table 2:** Statistical summary of social characteristics of the respondents.

Variable	Category	Frequency	Percentage (%)
Fuel Types	Mainly charcoal	68	24.4
	Mainly firewood	40	14.3
	Firewood and charcoal sometimes	98	24.7
	Mainly coal	12	4.3
	Liquefied petroleum gas (LPG)	90	32.3
Occupation	Civil servant	60	21.5
	Farming	91	32.6
	Own business	33	11.8
	Trading	94	33.7
Size of Apartment	Less than 4 rooms	185	66.3
	4 rooms and above	94	33.7
Gender	Male	146	52.3
	Female	133	47.7
Prefered Fuels If Given A Choice	LPG	138	49.5
	Charcoal	40	14.3
	Firewood	56	20.1
	Wood pellets	18	6.5
	Coal	27	9.7

**Table 3:** Households’ clean cooking (LPG in our case) awareness among participants.

Variable	Category	Frequency	Percentage (%)
**Source of Information**	No information	74	26.5
	Internet	11	3.9
	Radio	79	28.3
	Television	66	23.7
	Mobilizers	49	17.6
**Training**	Yes	64	22.9
	No	215	72.6

**Table 4:** Multinomial logit analysis for charcoal and liquefied petroleum gas (LPG) as compared to firewood.

Variables	Coef.	Std. Err.	z	*P* > z	Coef.	Std. Err.	z	*P* > z
Location	Charcoal				LPG			
Urban	0.7809	0.338	2.31	0.02	0.617	0.486	1.27	0.204
Household size	0.073	0.342	0.21	0.83	−0.040	0.463	−0.09	0.931
Education level	−0.541	0.337	−1.6	0.11	1.013	0.480	2.11	0.035
Migration	−0.277	0.376	−0.7	0.46	−0.141	0.563	−0.25	0.801
Occupation	−0.025	0.385	−0.1	0.95	−0.523	0.546	−0.96	0.337
Distance to fuel market	0.280	0.473	0.59	0.55	0.480	0.665	0.72	0.47
Gender								
Female	0.329	0.351	0.94	0.35	0.917	0.490	1.87	0.061
Who cooks								
any member of the family	−0.509	0.629	−0.8	0.42	1.310	0.825	1.59	0.112
Female member	−0.226	0.542	−0.4	0.68	−0.106	0.742	−0.14	0.886
Cooking time per day	−0.535	0.388	−1.4	0.17	−1.242	0.586	−2.12	0.034
Kitchen outside main house	14.005	451.386	0.03	0.98	11.577	451.386	0.03	0.98
Criteria for choosing fuel								
Availability	0.038	0.427	0.09	0.93	−0.316	0.561	−0.56	0.573
Emits less smoke	1.153	0.509	2.26	0.02	−1.316	0.931	−1.41	0.158
Quantity use per meal	−0.387	0.562	−0.7	0.49	−0.389	0.672	−0.58	0.562
Time saving	0.504	0.492	1.02	0.31	−0.309	0.733	−0.42	0.673
Credit use	0.718	0.364	1.97	0.05	2.272	0.477	4.76	0.00
Monthly income	0.602	0.324	1.86	0.06	0.758	0.446	1.7	0.09
Cooking monthly expenses	−0.017	0.339	−0.1	0.96	0.544	0.4599273	1.18	0.236
Size of apartment	0.014	0.356	0.04	0.97	1.062	0.491	2.16	0.031
Training	0.0419	0.404	0.1	0.92	0.928	0.521	1.78	0.075
_cons	−14.252	451.388	0	0.98	−14.943	451.388	−0.03	0.974

Note: Multinomial logistic regression; Number of obs = 279; LR chi2 (68) = 154; Prob > chi2 = 0.000; Log likelihood = −219.88062, and pseudo-*R*^2^ = 0.26.

## Data Availability

The data presented in this study are available on request from the corresponding author. The data are not publicly available due to permissions.

## References

[1] Chattopadhyay M., Arimura T.H., Katayama H., Mari S., Yokoo H. (2017). Cooking fuel choices—Analysis of socio-economic and demographic factors in rural India. Environ. Sci..

[2] Twumasi M.A., Jiang Y., Ameyaw B., Danquah F.O., Acheampong M.O. (2020). The impact of credit accessibility on rural households clean cooking energy consumption: The case of Ghana. Energy Rep..

[3] Li G., Bai X., Huo S., Huang Z. (2020). Fast pyrolysis of LERDADEs for renewable biofuels. IET Renew. Power Gener..

[4] Karimu A., Mensah J.T., Adu G. (2016). Who Adopts LPG as the Main Cooking Fuel and Why? Empirical Evidence on Ghana Based on National Survey. World Dev..

[5] Li G., Lu Z., Zhang J., Li H., Zhou Y., Zayan A.M.I., Huang Z. (2020). Life cycle assessment of biofuel production from microalgae cultivated in anaerobic digested wastewater. Int. J. Agric. Biol. Eng..

[6] Li G., Ji F., Bai X., Zhou Y., Dong R., Huang Z. (2019). Comparative study on thermal cracking characteristics and bio-oil production from different microalgae using Py-GC/MS. Int. J. Agric. Biol. Eng..

[7] Francis M., Geofrey O., Gemma A. (2014). Determinants of Household’s Choice of Cooking Energy in Uganda.

[8] Dendup N., Arimura T.H. (2018). Information leverage: The adoption of clean cooking fuel in Bhutan. Energy Policy.

[9] Dagnachew A.G., Hof A.F., Lucas P., van Vuuren D.P. (2019). Scenario analysis for promoting clean cooking in Sub-Saharan Africa: Costs and benefits. Energy.

[10] Accinelli R.A., Leon-Abarca J.A. (2017). Solid fuel use is associated with anemia in children. Environ. Res..

[11] Clark M.L., Peel J.L., Balakrishnan K., Breysse P.N., Chillrud S.N., Naeher L.P., Rode C.E., Vette A.F., Balbus J.M. (2013). Health and household air pollution from solid fuel use: The need for improved exposure assessment. Environ. Health Perspect..

[12] McCarron A., Uny I., Caes L., Lucas S.E., Semple S., Ardrey J., Price H. (2020). Solid fuel users’ perceptions of household solid fuel use in low- and middle-income countries: A scoping review. Environ. Int..

[13] Vaccari M., Vitali F., Mazzù A. (2012). Improved cookstove as an appropriate technology for the Logone Valley (Chad—Cameroon): Analysis of fuel and cost savings. Renew. Energy.

[14] Karanja A., Gasparatos A. (2018). Adoption and impacts of clean bioenergy cookstoves in Kenya. Renew. Sustain. Energy Rev..

[15] Paudel U., Khatri U., Pant K.P. (2018). Understanding the determinants of household cooking fuel choice in Afghanistan: A multinomial logit estimation. Energy.

[16] Hood A.H. (2010). The Use of Liquified Petroleum Gas (LPG) in Sudan. United Nations Environment Programme. www.unep.org/sudan.

[17] FAO (2016). Fuel-Efficient Mud Stoves in Darfur, Sudan.

[18] Omer A. (2002). Overview of renewable energy sources in the Republic of the Sudan. Energy.

[19] Li G., Zhang J., Li H., Hu R., Yao X., Liu Y., Zhou Y., Lyu T. (2020). Towards high-quality biodiesel production from microalgae using original and anaerobically-digested livestock wastewater. Chemosphere.

[20] Naab F.Z., Abubakari Z., Ahmed A. (2019). The role of climate services in agricultural productivity in Ghana: The per-spectives of farmers and institutions. Clim. Serv..

[21] Nnaji C.E. (2012). Determinanats of household energy choices for cooking in rural areas: Evidence from Enugu State, Nigeria. Cont. J. Soc. Sci..

[22] Gould C.F., Schlesinger S.B., Molina E., Bejarano M.L., Valarezo A., Jack D.W. (2020). Household fuel mixes in peri-urban and rural Ecuador: Explaining the context of LPG, patterns of continued firewood use and the challenges of induction cooking. Energy Policy.

[23] Amoah S.T. (2019). Determinants of household’s choice of cooking energy in a global south city. Energy Build..

[24] Alem Y., Beyene A.D., Köhlin G., Mekonnen A. (2016). Modeling household cooking fuel choice: A panel multinomial logit approach. Energy Econ..

[25] Ouedraogo B. (2006). Household energy preferences for cooking in urban Ouagadougou, Burkina Faso. Energy Policy.

[26] Ado A., Darazo I.R. (2016). Determinants of fuels stacking behaviour among households in Bauchi Metropolis. Bus. Manag. Rev..

[27] Ifegbesan A.P., Rampedi I.T., Annegarn H.J. (2016). Nigerian households’ cooking energy use, determinants of choice, and some implications for human health and environmental sustainability. Habitat Int..

[28] Ma W., Zhou X., Renwick A. (2018). Impact of off-farm income on household energy expenditures in China: Implications for rural energy transition. Energy Policy.

[29] Nabukalu C., Gieré R. (2019). Charcoal as an energy resource: Global trade, production and socioeconomic practices observed. Resources.

[30] Mamvura T., Pahla G., Muzenda E. (2018). Torrefaction of waste biomass for application in energy production in South Africa. South Afr. J. Chem. Eng..

[31] Lambe F., Jürisoo M., Wanjiru H., Senyagwa J. Bringing Clean, Safe, Affordable Cooking Energy to Households across Africa: An Agenda for Action. Prepared by the Stockholm Environment Institute, Stockholm and Nairobi, for the New Climate Economy. http://newclimateeconomy.report/misc/working-papers.

[32] FAO (2017). The Charcoal Transition: Greening the Charcoal Value Chain to Mitigate Climate Change and Improve Local Livelihoods.

[33] Pamela J., Das I. (2018). Energy for Sustainable Development Implementation and scale-up of a biomass pellet and improved cookstove enterprise in Rwanda. Energy Sustain. Dev..

[34] Weber E.U., Johnson E.J. (2009). Mindful Judgment and Decision Making. Annu. Rev. Psychol..

[35] Tsourgiannis L., Eddison J., Warren M. (2008). Factors affecting the marketing channel choice of sheep and goat farmers in the region of east Macedonia in Greece regarding the distribution of their milk production. Small Rumin. Res..

[36] Greene W.H., Hensher D.A. (2007). Heteroscedastic control for random coefficients and error components in mixed logit. Transp. Res. Part E Logist. Transp. Rev..

[37] Cohen J. (1992). A Power Primer. Psychol. Bull..

[38] dair-Rohani H., AlMazroa M.A., Amann M., Anderson H.R., Andrews K.G. (2012). A comparative risk assessment of burden of disease and injury attributable to 67 risk factors and risk factor clusters in 21 regions, 1990–2010: A systematic analysis for the Global Burden of Disease Study 2010. Lancet.

[39] Laufer D., Schafer M. (2011). The implementation of Solar Home Systems as a poverty reduction strategy—A case study in Sri Lanka. Energy Sustain. Dev..

[40] Neto-Bradley A.P., Rangarajan R., Choudhary R., Bazaz A. (2021). A clustering approach to clean cooking transition pathways for low-income households in Bangalore. Sustain. Cities Soc..

[41] Baland J. (2018). Pranab Bardhan, Samuel Bowles. Inequality, Cooperation, and Environmental Sustainability.

[42] van Rooyen C., Stewart T., de Wet T. (2012). The impact of microfinance in sub-Saharan Africa: A systematic review of the evidence. World Dev..

[43] Atieno R. (2001). Formal and Informal Institutions’ Lending Policies and Access to Credit by Small-Scale Enterprises in Kenya: An Empirical Assessment.

[44] Makhura M.N., Kirsten J., Delgado C., Friesen D.K., Palmer A.F.E. (2004). Transaction costs and smallholder participation in the maize market in the Northern Province of South Africa. Integrated Approaches to Higher Maize Productivity in the New Millennium, Proceedings of the Seventh Eastern and Southern Africa Regional Maize Conference, Nairobi, Kenya, 5–11 February 2002.

[45] Omiti J.M., Otieno D.J., Nyanamba T.O., McCullough E.B. (2009). Factors influencing the intensity of market participation by smallholder farmers: A case study of rural and peri-urban areas of Kenya. Afr. J. Agric. Resour. Econ..

